# Immobilized Antibodies on Mercaptophenylboronic Acid Monolayers for Dual-Strategy Detection of 20S Proteasome

**DOI:** 10.3390/s21082702

**Published:** 2021-04-12

**Authors:** Madalina M. Barsan, Caroline G. Sanz, Melania Onea, Victor C. Diculescu

**Affiliations:** 1 National Institute of Materials Physics, Atomistilor 405A, 077125 Măgurele, Romania; madalina.barsan@infim.ro (M.M.B.); caroline.sanz@infim.ro (C.G.S.); melania.onea@infim.ro (M.O.); 2 Faculty of Physics, University of Bucharest, Atomistilor 405, 077125 Măgurele, Romania

**Keywords:** electrochemistry, immunosensor, oriented antibodies, mercaptophenylboronic acid, self-assembled monolayers, 20S proteasome, enzymatic activity, proteolysis, labeled antibody, alkaline phosphatase

## Abstract

A dual strategy for the electrochemical detection for 20S proteasome (20S) is proposed, based on the oriented immobilization of a capture monoclonal antibody (Abβ) on a self-assembled monolayer of 4-mercaptophenylboronic acid (4-MPBA) on gold electrodes, which led to the Au/4-MPBA/Abβ immunosensor. The methodology comprises the correlation of 20S concentration with (i) its proteolytic activity toward the Z-LLE-AMC substrate, using the Au/4-MPBA/Abβ/20S, and (ii) the enzymatic activity of an alkaline phosphatase (AlkP) from the AlkP-labeled secondary antibody (Ab_core_-AlkP), which involves the conversion of aminophenylphosphate to the electroactive aminophenol using Au/4-MPBA/Abβ/20S/Ab_core_-AlkP. The step-by-step construction of the immunosensor and the interactions at its surface were evaluated by surface plasmon resonance and gravimetric analysis with quartz crystal microbalance, showing a high affinity between both antibodies and 20S. Morphological analysis by scanning electron microscopy demonstrated a pattern of parallel lines upon immobilization of Abβ on 4-MPBA and morphological changes to a well-organized granular structure upon binding of 20S. A voltametric and impedimetric characterization was performed after each step in the immunosensor construction. The two detection strategies were evaluated. It was shown that the immunosensor responds linearly with 20S concentration in the range between 5 and 100 µg mL^−1^, which corresponds to proteasome levels in serum in the case of diverse pathological situations, and LoD values of 1.4 and 0.2 µg mL^−1^ were calculated for the detection strategies. The immunosensor was applied to the detection of 20S in serum samples with recovery values ranging from 101 to 103%.

## 1. Introduction

The proteasome is a large enzyme involved in the controlled degradation of misfolded and damaged proteins controlling vital processes, therefore playing a key role in many pathological processes, such as inflammation, autoimmunity, neurodegenerative diseases, and cancer. An intense proteasome activity has been observed in the case of cancerous cells [1], with several inhibitors being effective for cancer treatment [2,3,4]. On the other hand, in the case of neurodegenerative diseases, proteins prone to form aggregates (α-synuclein, β-amyloid peptide, polyglutamine) compromise the proteasome function and delay the degradation of other proteasome substrates [5].

The proteasome is mostly found in the cytoplasm and nucleus and exists to a lesser extent in human serum, known as circulatory proteasome (proteasome c), with a comparatively low specific activity. The concentration of circulating proteasomes has been found to be elevated in patients suffering from autoimmune diseases, several types of cancer, solid tumors, sepsis and trauma, and its level can be correlated with the disease state [6].

The proteasome was first detected by enzyme-linked immunosorbent assay (ELISA) in 1993 [7], and studies in the last two decades have linked its elevated level to the existence of several diseases [6,8], as mentioned above. One type of ELISA tests for proteasome c detection is based on three antibodies: a capture antibody, a detection antibody and a third antibody labeled with horseradish peroxidase enzyme (HRP); the chromogenic HRP substrate 3,3′, 5,5′-tetramethylbenzidine is monitored by spectrophotometry at 450 nm. This assay was successfully used in the detection of proteasome c in cases of ischemic heart disease [9] and in lung diseases [10]. Another proteasome c detection kit uses electro-chemiluminescence, in which the third antibody that allows quantification is labeled so as to generate an electro-chemiluminescent signal, and has been successfully used to detect proteasome c in patients with hepatocellular carcinoma [8]. Surface plasmon resonance (SPR) imaging has also been applied for 20S detection, based on the interaction of proteasome with immobilized inhibitors [11,12].

Electrochemical immunosensors have emerged recently as cost-effective and highly sensitive tools in clinical diagnosis for the detection of various key analytes [13,14,15,16,17]. The present study reports the development and application of an immunosensor for the electrochemical detection of 20S proteasome (20S). To our knowledge, only one electrochemical immunosensor for 20S has been reported previously, based on an electroactive polymer responsible for the detection principle, with the decrease in the polymer peak currents being correlated to the linkage of 20S to an immobilized 20S antibody [18].

The proposed architecture involves immobilization of a capture antibody (Abβ) specific for the β5 subunit of the 20S on an Au electrode previously modified with a self-assembled monolayer (SAM) of 4-mercaptophenylboronic acid (4-MPBA), leading to the Au/4-MPBA/Abβ immunosensor. The choice for this specific capture antibody is based on previous observations that Abβ binds to the 20S in a conformation that allows an easy access of the substrate to its catalytic core [19]. On the other hand, self-assembled monolayers (SAMs) are widely used to immobilize biorecognition elements on solid surfaces. The choice of 4-MPBA was the result of the finding that boronic acid derivatives have advantages in immobilizing the antibody through self-assembly reactions with the exposure of its antigen-binding site [20,21]. The interaction between Abβ and 4-MPBA makes use of the diol formation between the -OH groups of 4-MPBA and the carbohydrates in the Fc region of the antibody.

In the above-described configuration, the self-assembled layer of 4-MPBA with Abβ on gold electrode leads to the Au/4-MPBA/Abβ immunosensor, which allows binding of 20S with high affinity. A dual electrochemical detection strategy for 20S detection at the Au/4-MPBA/Abβ immunosensor is proposed.

The first strategy comprises the correlation of 20S concentration with its proteolytic activity when bound to Abβ. The Au/4-MPBA/Abβ/20S obtained in this step allows for investigation of the 20S activity by fixed potential amperometry, as perfected in a preceding study [19], with the detection principle based on the oxidation of an electroactive probe released from the enzyme’s substrate upon the proteolysis [22,23]. The 20S-catalyzed proteolysis of the specific substrate is dependent on 20S concentration at the surface of the Au/4-MPBA/Abβ/20S, allowing its quantification through the kinetic parameters of the 20S enzymatic reaction.

The second strategy involves a detection antibody (Ab_core_) labeled with the enzyme alkaline phosphatase (AlkP), which binds to 20S at the surface of the Au/4-MPBA/Abβ/20S. Ab_core_ is specific to the core subunits of 20S and was chosen to enable maximum binding efficiency to the immobilized 20S. The AlkP enzyme allows the conversion of aminophenylphosphate (AmPhP) to the electroactive aminophenol (AmPh) [24,25,26], which can be electrochemically monitored at low potential values [24,25]. The Ab_core_-AlkP complex binding is dependent on 20S concentration at the surface of the Au/4-MPBA/Abβ/20S, allowing its quantification through the kinetic parameters of the AlkP enzymatic reaction.

The immunosensor construction and interaction with 20S and Ab_core_-AlkP were monitored by surface plasmon resonance and quartz crystal microbalance and characterized morphologically by scanning electron microscopy. Cyclic voltammetry and electrochemical impedance spectroscopy were also used for immunosensor characterization and the interactions taking place at the surface. Fixed potential amperometry was applied for the electrochemical quantification of 20S in the above-described configurations.

## 2. Materials and Methods

### 2.1. Reagents and Solutions

The reagents were of analytical grade and were used without further purification. Millipore Milli-Q nanopure water (resistivity ≥ 18 MΩ cm) was used for the preparation of all solutions.

Proteasome 20S (human) and 20S β5 subunit (human) monoclonal antibody (Abβ), 20S core subunits polyclonal antibody (Ab_core_) and 20S substrate carbobenzoxy(Z)-Leu(L)-Leu(L)-Glu(E)-7-amino-4-methylcoumarin (AMC) of caspase activity were from Enzo Life Sciences. The 4-mercaptophenylboronic acid, AmPhP, AmPh, Tris/HCl, KCl, NaCl, MgCl_2_, SDS, EDC, NHS, PBS with 0.1% Tween, and Fetal Bovine Serum with ≤10 EU/mL endotoxins were from Sigma-Aldrich. Alkaline Phosphatase Labeling Kit-NH_2_ was from Abnova (St. Louis, MO, USA). The labeling of Ab_core_ with AlkP was done in agreement with the instructions in the labeling kit manual.

Stock solutions of Z-LLE-AMC, AmPh and AmPhP were prepared in dimethyl sulfoxide (DMSO). Stock solutions of 20S proteasome were prepared in water and kept at −20 °C until further utilization.

The proteasome assay buffer (PAB) pH = 7.5 contained 50 mM Tris/HCl, 25 mM KCl, 10 mM NaCl, 1 mM MgCl_2_ and 100 µM SDS.

### 2.2. Instrumentation

The quartz crystal microbalance Model QCM922 from Princeton Applied Research was used with an Au quartz crystal with a 9 MHz central frequency.

Surface plasmon resonance (SPR) experiments were performed on a Reichert SR7500DC SPR spectrometer (Reichert Technologies Life Sciences, Buffalo, NY, USA) equipped with a SR7500 250 μL syringe pump and a SR8100 autosampler with a 100 μL injection loop. The SPR sensors were prepared on glass slides with the deposition of 1 nm thick Ti layer by RF sputtering and a 50 nm Au layer by vacuum thermal evaporation. The Au chip was chemically modified with 4-MBPA as described in Section 2.3. Solutions of Abβ, Ab_core_-AlkP and 20S were prepared in phosphate buffer (PB), and full loop injections were performed at 25 °C with an infuse rate of 5 μL min^−1^. The running buffer was PBS with 0.1% Tween.

A Zeiss Evo 50 XVP SEM (Carl Zeiss NTS, LLC, Oberkochen, Germany) was used for the morphological characterization of the electrode surface. The probes were deposited on Si/SiO_2_/Ti/Au electrodes, with the 200 nm Au layer being deposited by thermal evaporation. The SEM images were acquired at different magnifications.

The electrochemical measurements were performed in a three-electrode configuration, with a silver/silver chloride (Ag/AgCl) electrode as a reference, a platinum wire as counter and an Au bulk electrode as the working electrode (1.6 mm diameter, 0.02 cm^2^). The electrochemical cell contained 1 mL electrolyte. All the electrochemical measurements were carried out with a IVIUM (Eindhoven, The Netherlands) potentiostat-galvanostat.

For fixed potential amperometry, the current was sampled every 0.2 s at potential values indicated in the text.

Electrochemical impedance spectroscopy (EIS) experiments were carried out using an rms perturbation of 10 mV applied over the frequency range 100 kHz–0.1 Hz, with 10 frequency values per frequency decade. The spectra were recorded at applied potential values indicated in the text and fitted with the ZView 3.2 software (Scribner Associates Inc., Southern Pines, NC, USA).

### 2.3. Preparation of the Immunosensor

The Au electrode surface was cleaned first by mechanical polishing using diamond spray, particle size 3 μm (Kemet International Ltd., Kent, UK), followed by chemical cleaning by recording 10 cyclic voltammograms in 50 mM H_2_SO_4_.

The Au was first modified with 4-MPBA by immersing the electrode in a 20 mM ethanolic solution for 24 h. The Au/4-MPBA electrode was then rinsed sequentially with ethanol and water and dried. 2 µL of 1 mg mL^−1^ capture antibody (Abβ) was immobilized in the presence of 1 µL of 4% *w*/*v* 1-Ethyl-3-(3-dimethylaminopropyl)carbodiimide (EDC) in dimethylformamide (DMF) and 1 µL 1% *w*/*v* N-hydroxy succinimide (NHS) in water. The Au/4-MPBA/Abβ immunosensor was allowed to dry in air for 2 h, and the reagent excess was washed by immersion in water.

For the Quartz Crystal Microbalance (QCM) experiments, a similar procedure was used, adapting the required volumes for the modification to the geometric area of the crystal, 0.1963 cm^2^.

### 2.4. Procedure for 20S Detection

The Au/4-MPBA/Abβ immunosensor was incubated for 30 min in aqueous 20S solutions of 0.05, 0.5, 5, 50, 100, 250 and 1000 µg mL^−1^. The excess was removed by immersion in water.

In the first procedure, the Au/4-MPBA/Abβ/20S was used for 20S activity studies toward the proteolysis of Z-LLE-AMC. Injection of 5 µL Z-LLE-AMC was performed into the PAB-containing electrochemical cell, with final concentrations depicted in the corresponding figures. The measurements were carried out at +0.80 V by fixed potential amperometry.

In the second procedure, the Au/4-MPBA/Abβ/20S was incubated in the detection antibody Ab_core_-AlkP for 1 h and then used for AlkP activity studies towards the dephosphorylation of AmPhP. Injection of 10 µL AmPhP was performed into the electrochemical cell containing 0.1 M PB, pH = 8.5, with final concentrations depicted in the corresponding figures. The measurements were carried out at +0.20 V by fixed potential amperometry.

**Scheme 1:**
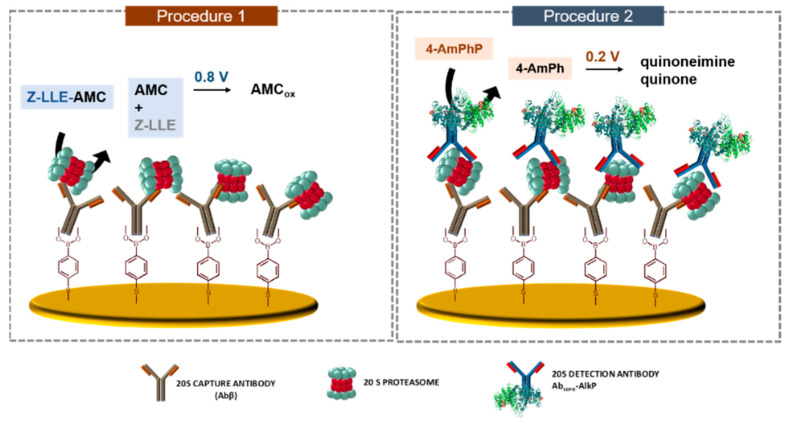
20S immunosensor architecture following Procedures 1 (Au/4-MPBA/Abβ/20S) and 2 (Au/4-MPBA/Abβ/20S/Ab_core_-AlkP).

The schematic representation of the immunosensor and the assembly with 20S and Ab_core_-AlkP is presented in Scheme 1.

### 2.5. Real Sample Analysis

The fetal bovine serum sample was diluted 10 times with water. A 50 µg mL^−1^ 20S solution was prepared in the diluted fetal bovine serum.

## 3. Results and Discussion

### 3.1. Monitoring the Step-by-Step Construction

#### 3.1.1. Surface Plasmon Resonance

**Figure 1:**
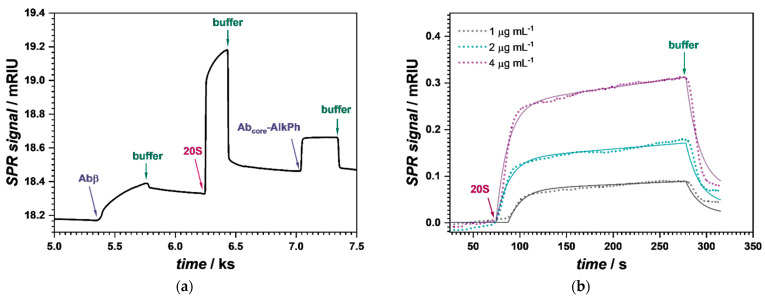
SPR signal recorded (**a**) at Au/4-MPBA after consecutive injections of Abβ antibody, 10 µg mL^−1^ 20S and Ab_core_-AlkP and (**b**) at Au/4-MPBA/Abβ after consecutive injections of 1, 2 and 4 µg mL^−1^ of 20S.

The construction of the sensing layer was also investigated by SPR after consecutive injections of each immunosensor component (Abβ, 20S and Ab_core_-AlkP) in running buffer (Figure 1a) in order to evaluate the changes in the vicinity of the Au/4-MPBA sensor due to the binding processes. Increase of the SPR signal was observed during association/interaction processes after the injection of each component. For the dissociation steps when only the buffer was flowed over the sensor surface, a small decrease—which eventually reached stable values—was observed. The baseline shifted to higher values after the adsorption/dissociation phase, which indicates the incorporation of each element on the Au/4-MPBA sensor.

The analysis of the curve referent to the Abβ injection allowed the determination of the association and dissociation rate constants *k*_a_ = 3.1 × 10^4^ M^−1^ s^−1^ and *k*_d_ = 1.6 × 10^−3^ s^−1^; thus resulting in a low-value equilibrium dissociation constant *K*_D_ = 5.3 × 10^−8^ M, showing the stability of the 4-MPBA/Abβ complex.

A similar experiment was conducted, and different concentrations of 20S (1, 2 and 4 µg/mL) were consecutively injected over the Au/4-MPBA/Abβ immunosensor surface (Figure 1b). The increase of the SPR signal with increasing 20S concentrations was observed during both association and dissociation phases. The analysis of the curve was carried out by fitting the experimental data with a “One-to-One Two-State” model, characterized by a weak initial interaction event followed by a stronger type after the binding partners have been in complex for some time, which returned a value of *K*_D_ = 6.01 × 10^−14^ M, showing a very high stability of the MPBA/Abβ/20S complex. Similarly, the equilibrium dissociation constant *K*_D_ = 1.32 × 10^−9^ M for the Ab_core_-AlkP binding to the immobilized 20S proteasome was determined.

#### 3.1.2. Gravimetric Analysis

In the construction of immunosensors, it has been shown that the immobilization strategies employing the chemical linkage of the first antibody can be random or oriented (or site-specific), with the latter allowing a better availability of the antibody binding sites for the interaction with the antigen, which eventually can lead to a significant enhancement in the immunosensor signal response [27]. Strategies in improving the chemical linkage of the antibody, avoiding the random blockages of the binding site, involve the use of boronic acid derivatives, which allow a favorable orientation-controlled arrangement of antibodies with the exposure of its antigen-binding site [21]. The chemical adsorption of 4-MPBA was monitored using a QCM, and a total mass of 30 ng was determined. The dimension of one molecule of 4-MPBA was considered to be ~0.4 nm^2^ and served to calculate the area of one monolayer of 4-MPBA as 0.24 cm^2^, 20% higher than the geometrical area of the AuQC. The results indicate the formation of a 4-MPBA SAM on Au, which serves to anchor the capture antibody (Abβ) on the electrode surface. A total mass of 233.3 ng per cm^2^ of the immobilized Abβ was determined, which is more than twice that reported for similar immobilization strategies [20].

The following step concerns the immobilization of the 20S, which was done by the immersion of AuQC/4-MPBA/Abβ in solutions of 20S with increasing concentrations: 5, 50, 100, and 250 µg mL^−1^, with the calculated mass of bounded 20S of 1.68, 11.54, 24 and 33 ng, respectively, and a total mass of 70.2 ng 20S. The results indicate a molar ratio of 17.7:1 Abβ:20S. Considering that the size of the Abβ antibody is ≈6 times smaller than 20S, the Abβ is organized in a densely packed film, therefore limiting a higher 20S binding.

### 3.2. Characterization

#### 3.2.1. Morphological—Scanning Electron Microscopy

**Figure 2:**
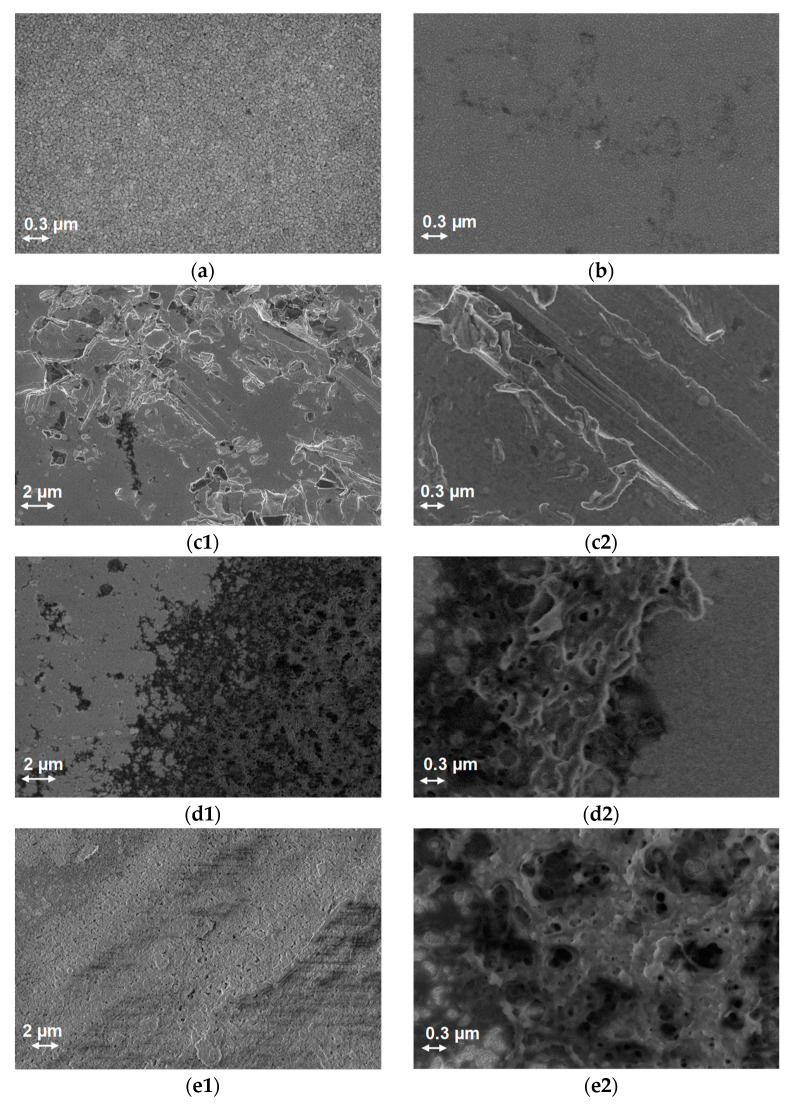
SEM images of (**a**) Au and (**b**) Au/4-MBPA at 50 × 103 magnification; (**c**) Au/4-MPBA/Abβ, (**d**) Au/4-MPBA/Abβ/20S and (**e**) Au/4-MPBA/ Abβ/20S/Abcore-AlkP at (**1**) 10 × 103 and (**2**) 50 × 103 magnification.

SEM images of Au, Au/4-MPBA, Au/4-MPBA/Abβ, Au/4-MPBA/Abβ/20S and Au/4-MPBA/Abβ/20S/Ab_core_-AlkP are shown in Figure 2. The typical granular structure of Au deposited by thermal evaporation on Si/SiO_2_/Ti substrates (Figure 2a) does not change significantly upon modification with the SAM of 4-MPBA, with a thin 4-MPBA layer uniformly coating the entire electrode surface (Figure 2b). Abβ is immobilized at Au/4-MPBA following a pattern of parallel lines, as is clearly shown in Figure 2c, with an organization in micrometer-size clusters. The immobilization of 20S leads to a complete change in the surface morphology, with the appearance of well-organized granular structures covering in multiple layers the whole surface (Figure 2d), as expected due to the nanometer cylindric shape of 20S [28]. This porosity given by the deposition of 20S is then flattened out upon adsorption of Ab_core_-AlkP, as shown in Figure 2e1, with some granular features being maintained in some areas, exemplified in Figure 2e2.

#### 3.2.2. Electrochemical

- Cyclic voltammetry

**Figure 3:**
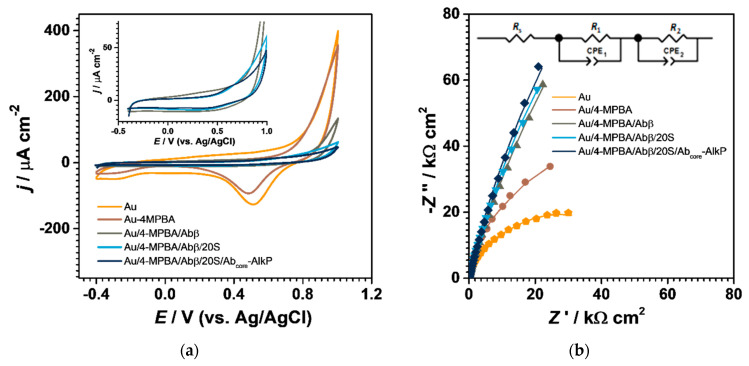
(**a**) Cyclic voltammetry at 50 mV s^−1^ (2nd cycle); (**b**) Nyquist diagram of EIS, recorded in deoxygenated 0.1 M PB pH 8.5 during the step-by-step assembly of Au/4-MPBA/Abβ/20S/Ab_core_-AlkP.

The electrode surface was investigated by cyclic voltammetry (CV) after each modification step. The voltammograms recorded with Au, Au/4-MPBA, Au/4-MPBA/Abβ, Au/4-MPBA/Abβ/20S and Au/4-MPBA/Abβ/20S/Ab_core_-AlkP in deoxygenated 0.1 M PB, pH 8.5, are shown in Figure 3a.

In the potential region between −0.40 and +1.0 V, the Au electrode exhibited its typical redox behavior. Upon modification with 4-MPBA, one oxidation peak is visible at +0.45 V in the first cycle [29], but is no longer seen on subsequent cycles since MBPA oxidation is irreversible. In addition, the incorporation of 4-MPBA induces a decrease of the capacitive contribution characteristic to organic adsorption, attributed to the low dielectric constant [30]; the capacitance remained unchanged upon consecutive cycling. 

Upon further modifications with the Abβ, Abβ/20S and Abβ/20S/Ab_core_-AlkP, the capacitive current continues to decrease, with the complete blockage of the Au electrode surface; no redox behavior of Au was observed.

- Electrochemical impedance spectroscopy (EIS)

EIS was performed to characterize the surface of the immunosensor and to evaluate the influence of each component during the step-by-step immunosensor assembly. Impedance spectra obtained in deoxygenated 0.1 M PB pH 8.5 at −0.20 V (Figure 3b) were fitted to an equivalent electrical circuit (inset of Figure 3b) that comprises the cell resistance (*R*_s_) in series with two *RC* combinations, one for the electrode/film and one for the film/solution interface, with the first being used to model the phenomena between 65,000 to 100 and the second for 100 to 0.1 Hz, respectively. Constant phase element (CPE) was used, considering the irregularities and non-uniformities of both solid/solid and solid/solution interfaces. The *R*_s_ value was ≈200 Ω, which represents the ohmic drop and resistance caused by the electrical contacts and solution.

At the bare Au electrode, the two *R*CPEs were assigned to the Au/Au oxide and Au oxide/solution interfaces; for the other configurations, they were assigned to the Au/film and film/solution interfaces. 

The *R*CPE*2* values assigned to the film/solution interface changed in agreement with the CV experiments (Table 1). A similar Nyquist profile for an alkanethiol modified Au was observed in [31]. Upon electrode modification with 4-MPBA, *R*_2_ value increased by a factor of 2 and increased with one order of magnitude upon addition of the first proteic layer, the Abβ. Upon consecutive addition of 20S and Ab_core_-AlkP, the film resistance continues to increase due to the increase in film thickness. On the other side, the CPE2 values decrease gradually during the step-by-step assembly, as expected. The CPE2 value of the Au/4-MBPA is higher than expected for SAM of alkanethiol films, which can be explained considering (1) 4-MPBA is a larger molecule compared to alkanethiols, allowing ions to penetrate within the film, (2) there are therefore unmodified areas of Au, sustained by the CV profile in Figure 3a, where the redox behavior of Au is still visible after the SAM assembly and (3) a reduction of film crystallinity, which induces an increase in ionic permeability of the film [31]. Note that *α*_2_ values are close to 1, and 1 in the case of 4-MBPA, reflecting the organization of the films.

### 3.3. Applications—Quantification of 20S Proteasome

The quantification of 20S at the surface of the Au/4-MPBA/Abβ immunosensor was done employing two different strategies, including (i) using the Au/4-MPBA/Abβ/20S to evaluate/correlate the 20S enzymatic activity with its concentration and (ii) using the Au/4-MPBA/Abβ/20S/Ab_core_-AlkP, which correlates directly the amount of AlkP-labeled detection antibody with the 20S at the immunosensor surface using AlkP activity.

#### 3.3.1. Au/4-MPBA/Abβ/20S

**Scheme 2:**
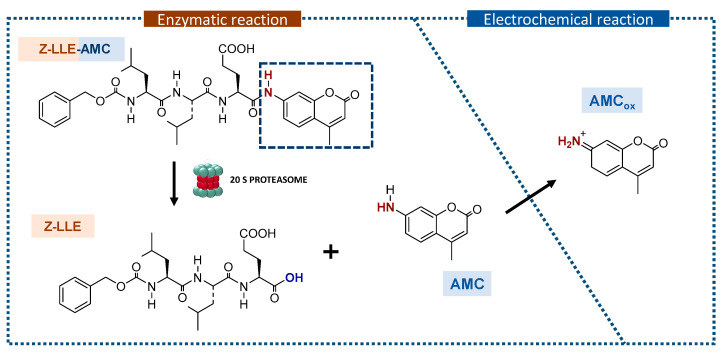
The enzymatic cleavage of Z-LLE-AMC by 20S coupled with the electrochemical AMC oxidation [19].

This strategy comprises the correlation of 20S concentration with its proteolytic activity on the Z-LLE-AMC substrate, designed to undergo proteolysis by 20S at the peptide bond between the Glu(E) amino acid and the AMC moiety, which leads to the release of the electroactive AMC molecule (Scheme 2). It has been previously shown that the release of AMC upon proteolysis produces an anodic peak characteristic to the amine moiety of free AMC molecules (*E*_pa_ ~ +0.80 V vs. Ag/AgCl at pH ~ 7.0), which does not occur when the molecule is bound to the peptide substrate [19,22,23]. The 20S-catalyzed proteolysis of the Z-LLE-AMC substrate is dependent on the 20S amount at the surface of the Au/4-MPBA/Abβ/20S immunosensor, allowing its quantification through the kinetic parameters of the 20S enzymatic reaction.

**Figure 4:**
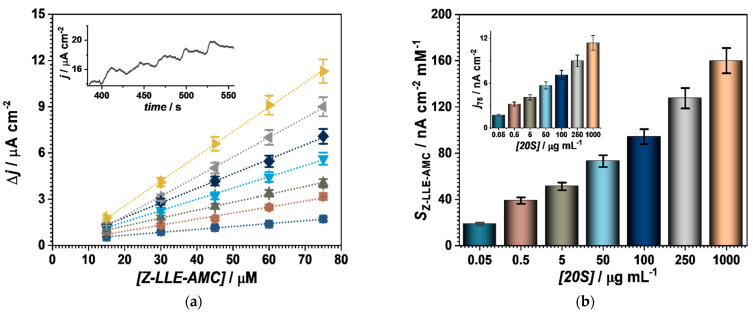

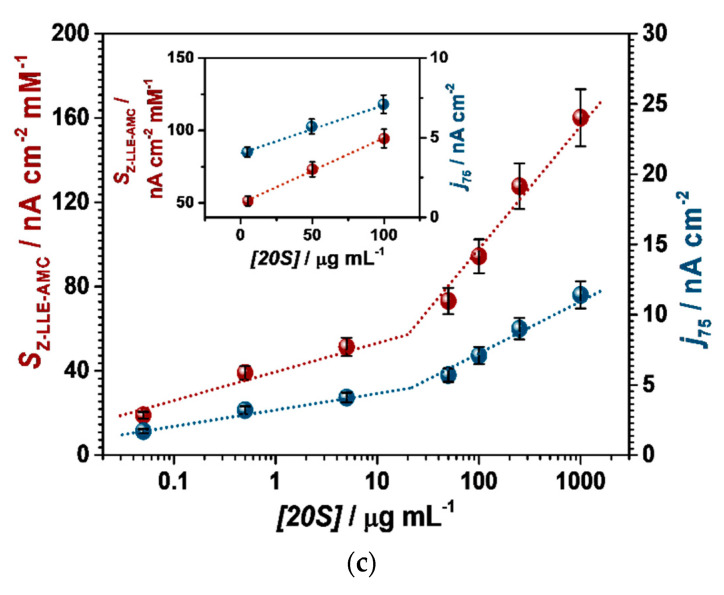
(**a**) Calibration curves of 20S proteasome activity upon injections of Z-LLE-AMC substrate at Au/4-MPBA/Abβ/20S containing different 20S concentrations: 0.05, 0.5, 5, 50, 100, 250 and 1000 µg mL^−1^; the inset presents a typical CA response at +0.80 V; (**b**) dependence of *S*_Z-LLE-AMC_ on 20S concentration; (**c**) dependence of both *S*_Z-LLE-AMC_ and *j_75_* on log *[20S]*; inset represents the linear dependence of *S*_Z-LLE-AMC_ and *j_75_* with *[20S]* between 5 and 100 µg mL^−1^.

Au/4-MPBA/Abβ/20S containing different 20S concentrations—0.05, 0.5, 5, 50, 100, 250 and 1000 µg mL^−1^—were subjected to chronoamperometry (CA) experiments recorded with successive injections of the peptide Z-LLE-AMC in the concentration range from 12.5 to 75 µM; the corresponding calibration plots are presented in Figure 4a. The inset contains a typical CA response, with an anodic change in current upon the peptide Z-LLE-AMC injection due to the oxidation of AMC released by the 20S enzymatic action.

The analytical parameters obtained from the calibration plots in Figure 4a are presented in Table S1. As observed, the sensitivity of the Au/4-MPBA/Abβ/20S for proteolysis of the Z-LLE-AMC substrate (*S*_Z-LLE-AMC_) increases with an increase in the amount of immobilized 20S, ranging from 18.9 ± 1.3 to 160.1 ± 10.3 nA cm^−2^ mM^−1^ for 0.05 and 1000 µg mL^−1^ 20S, respectively (Figure 4b). The current recorded after injection of 75 µM Z-LLE-AMC (*j*_75_) increases with an increase of the immobilized 20S (inset of Figure 4b), from 1.7 ± 0.2 nA cm^−2^ to 11.4 nA ± 0.9 nA cm^−2^ for 0.05 and 1000 µg mL^−1^ 20S, respectively. 

Two linearity domains were observed for both *S*_Z-LLE-AMC_ and *j*_75_, with a decimal logarithm of 20S concentration (Figure 4c). The first domain was up to approximately 20 µg mL^−1^, while the second domain comprises the concentration interval from approximately 20 up to 1000 µg mL^−1^ and showed a slope value much higher than for the low concentration range (Table S2). The two linear domains in the logarithmic calibration plots in Figure 4c can be explained by different stabilities of the 20S-antibody complexes, with the weaker interaction taking place for low concentrations of 20S, which grows stronger with increasing enzyme concentration, as previously demonstrated by SPR measurements (see Section 3.1.1).

In the concentration range 5–100 µg mL^−1^ 20S, the *S*_Z-LLE-AMC_ and *j*_75_ increase linearly with the 20S amount (inset of Figure 4c), following Equations (1) and (2), respectively:

*S*_Z-LLE-AMC_/nA cm^−2^ mM^−1^ = 49.6 + 0.5 × *[20S]*/µg mL^−1^ (1)

*J*_75_*/*nA cm^−2^ = 4.12 + 0.03 × *[20S]*/µg mL^−1^ (2)

This evidences not only the applicability of Au/4-MPBA/Abβ/20S for the quantification of 20S but also for the evaluation of 20S activity. It is important to mention that this concentration range corresponds to proteasome c levels in serum in the case of diverse pathological situations [6,32]. The LoDs was calculated to be 1.4 and 14.0 µg mL^−1^ 20S, respectively. The immunosensor sensitivity was higher by a factor of almost 10 when compared with a immunosensor based on the Abβ immobilized by crosslinking on a GCE [19] and by a factor of 2 on Au (data not shown), proving the importance of the use of 4-MPBA for the oriented immobilization of Abβ.

RSD values were determined by considering three different immunosensors, with values between 7.4 and 8.8%, indicating a good reproducibility in the fabrication of the Au/4-MPBA/Abβ/20S immunosensor.

Repeatability was determined by recording three-point calibration plots at Au/4-MPBA/Abβ/20S, with RSD values lower than 4% for the sensitivity values.

The immunosensor operational stability was evaluated for a period of seven days, being tested each day for one Z-LLE-AMC concentration. The immunosensor response had a decrease of only 10% after seven days.

#### 3.3.2. Au/4-MPBA/Abβ/20S/Ab_core_-AlkP

Within this strategy, the AlkP enzyme is used in synergy with the AmPhP electrochemically inert substrate, which under enzymatic action is dephosphorylated and transformed into AmPh, a product that undergoes oxidation at the electrode surface. The AlkP is brought to the Au/4-MPBA/Abβ/20S surface through Ab_core_, and the amount of the Ab_core_-AlkP is proportional to the 20S, allowing its quantification at the surface of Au/4-MPBA/Abβ/20S/Ab_core_-AlkP through the kinetic parameters of the AlkP enzymatic reaction.

**Scheme 3:**
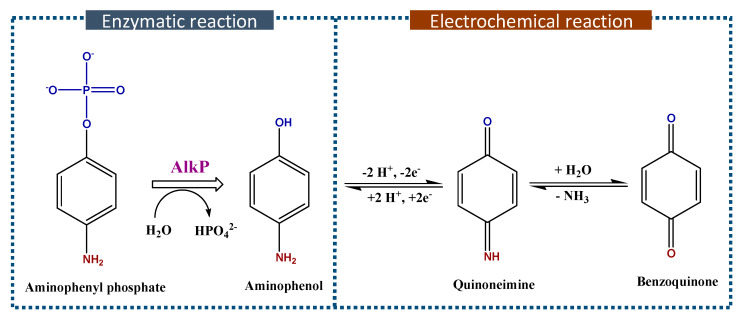
The enzymatic dephosphorylation of AmPhP by AlkP coupled with the electrochemical oxidation of AmPh [33].

The electrochemical detection of AmPh was first performed at a bare Au, by CV (Figure S1a) and fixed potential amperometry with an applied potential of +0.20 V (Figure S1b). As shown in Figure S1a, AmPh undergoes a reversible electrochemical reaction (Scheme 3), and the scan rate study indicates a diffusion controlled process (data not shown). Considering the CV data, fixed potential CA measurements were performed at +0.20 V, a potential value that allows its oxidation. The typical response is shown in Figure S1b, with an anodic change in current following AmPh injection. The bare Au electrode had a sensitivity of 164.3 µA cm^−2^ mM^−1^ with a detection limit of 7.7 µM towards AmPh detection, similar to that reported in [24].

**Figure 5:**
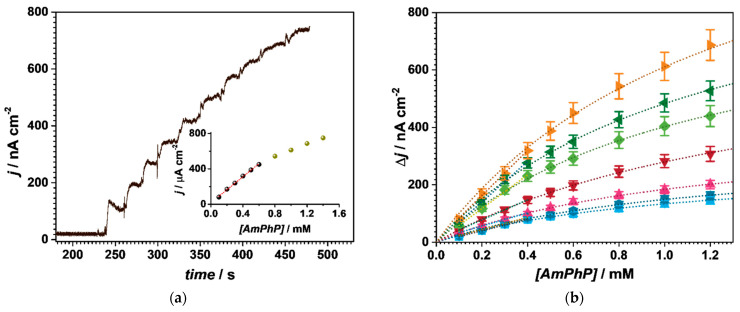

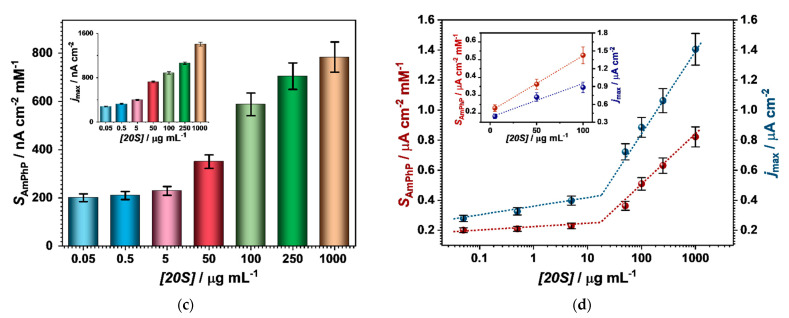
(**a**) CA response recorded at Au/4-MPBA/Abβ/20S/Ab_core_-AlkP (with 1000 µg mL^−1^ 20S) at +0.20 V upon successive injections of AmPhP; the inset is the corresponding calibration curve; (**b**) calibration curves for different 20S concentrations and non-linear fitting with the Michaelis Menten model (fitting parameters *j*_max_ and *K*_m_ provided in Table S4); (**c**) *S*_AmPhP_ values for different 20S concentrations (the inset represents *j_max_* values for different 20S concentrations); (**d**) dependence of both *S*_AmPhP_ and *j*_max_ on log*[20S]*; inset represents the linear dependence of *S*_AmPhP_ and *j*_max_ with *[20S]* between 5 and 100 µg mL^−1^.

The Au/4-MPBA/Abβ/20S/Ab_core_-AlkP was tested similarly in CA experiments at +0.20 V, with the injection of AlkP substrates, AmPhP, the enzymatic reaction product being AmPh. A typical CA response of the immunosensor containing 1000 ng mL^−1^ 20S is shown in Figure 5a (inset is the corresponding calibration curve with the linear domain).

As expected, an anodic change in current is recorded upon injection of AmPhP (Figure 5a) due to the oxidation of the enzyme reaction product AmPh, with a saturation for concentrations higher than 0.6 mM AmPhP. Calibration curves were constructed for the immunosensors containing 20S concentrations of 0.05, 0.5, 5, 50, 100, 250 and 1000 µg mL^−1^ and are displayed in Figure 5b.

The immunosensors presented a typical enzymatic response, with saturation for concentrations of AmPhP substrate higher than 0.4 mM. For concentration values below 0.4 mM AmPhP, the curves displayed a linear range, and the sensitivity of the Au/4-MPBA/Abβ/20S/Ab_core_-AlkP immunosensor for dephosphorylation of AmPhP (*S*_AmPhP_) increased from 200.3 ± 2.1 to 784.5 ± 8.7 nA cm^−2^ mM^−1^, with increasing proteasome concentration (Figure 5c and Table S3).

For the whole concentration range, the curves were fitted using the adapted Michalis Menten equation, in which *j*_max_ substitutes for *V*_max_. The obtained enzymatic parameters are presented in Table S4. As expected, the *K*_m_ values remain constant with the variation of 20S in the immunosensor, with a slightly higher value when compared to that of the enzyme in solution [34]. The value of *j_m_*_ax_ increases with the increase of the immobilized 20S, from 280.3 ± 3.1 nA cm^−2^ to 1400.6 ± 11.8 nA cm^−2^ (inset of Figure 5c).

Two linearity domains were observed for both *S*_AmPhP_ and *j*_max_, with decimal logarithm of 20S concentration (Figure 5d) similar to that observed during the electrochemical quantification of 20S at the Au/4-MPBA/Abβ/20S (see Section 3.3.1). The first domain was up to approximately 20 µg mL^−1^, while the second domain comprises the concentration interval from 20 up to 1000 µg mL^−1^ and showed a slope value much higher that for the low concentration range (Table S5). However, the immunosensor presented a linear dependence of both *S*_AmPhP_ and *j*_max_ for the 20S concentration range between 5 and 100 µg mL^−1^ (inset of Figure 5d), which corresponds to the concentrations of proteasome c in serum in the case of diverse pathological situations [6,32]. The relationships are

*S*_AmPhP_/nA cm^−2^ mM^−1^ = 210.2 + 3.1 × *[20S]*/µg mL^−1^ (3)

*j*_max_*/*nA cm^−2^ = 383.5 + 5.7 × *[20S]*/µg mL^−1^ (4)

resulting in an LoD of 0.2 and 0.6 µg mL^−1^, respectively.

The RSD values calculated for three different immunosensors for each 20S concentration were below 8.8%, which indicates a reproducible analytical method employing the herein developed immunosensors for the detection of 20S. Repeatability studies were also done, and the RSD calculated for the three experiments was lower than 5%. The immunosensor maintained 95% of the initial response after two weeks of storage in air at 4 °C.

Specificity studies were performed with the protein bovine serum albumin, and the changes in current values at a concentration of 5, 50 and 100 µg mL^−1^ were close to 0 (for addition of 0.1 mM AmPhP). A test was made with 5 µg mL^−1^ BSA, immobilized together with 50 µg mL^−1^ 20S in the immunosensor architecture, and results were compared with the one containing the 20S alone. The ratio of the current with interfering substance to pure 20S was 1.01, which shows no interference from BSA.

#### 3.3.3. Recovery

Real sample analysis of the proteasome immunosensor fetal bovine serum sample (*n* = 3) was made by preparing a proteasome solution of 50 µg mL^−1^ in the fetal bovine serum. For this, both 20S detection methods were employed, using the Au/4-MPBA/Abβ/20S and the Au/4-MPBA/Abβ/20S/Ab_core_-AlkP. The matrix effect of the probe was evaluated by comparing *S*_Z-LLE-AMC_ and *S*_AmPhP_ in serum with those obtained using standard 20S solutions (Tables S1a and S2a, respectively) for 50 µg mL^−1^ 20S. The *S*_Z-LLE-AMC_ value at Au/4-MPBA/Abβ/20S was 75.2 ± 7.1 (nA cm^−2^ mM^−1^) and the *S*_AmPhP_ value at Au/4-MPBA/Abβ/20S/Ab_core_-AlkP was 366.3 ± 18.0 (nA cm^−2^ mM^−1^). The recovery values were 103% and 101%, respectively.

#### 3.3.4. Comparison with Other AlkP Based Electrochemical Immunosensors

Most of the herein cited [24,35,36,37,38,39,40] electrochemical immunosensors based on AlkP labeled antibodies have a more complex architecture, based on polymers [24,38], magnetic beads [39] or carbon nanomaterials [40] (Table 2). Nonetheless, these examples involve different substrates for the AlkP enzyme, which has a strong effect on the detection limit of the reaction products due to the enzyme affinity and kinetic parameters for each individual substrate.

A comparison between biosensors for different antigens is not straightforward and must take into account the simplicity of the electrode architecture, the application and the relevance of the dynamic ranges for a relevant biomarker. The main two advantages of the present immunosensor are its simple architecture and the linear dependency over the concentration range 5–100 µg mL^−1^, which corresponds to proteasome levels in serum in the case of diverse pathological situations, all these making it cost-effective and easy to fabricate.

#### 3.3.5. Comparison with Other 20S Detection Methods

The performance of the present immunosensor was compared to previous reports related to detection methodologies of 20S proteasome (Table 3). In relation to the SPR method, which enabled the detection of proteasome through its interaction with inhibitors [11,12], similar detection limits are observed; however, the Au/4-MPBA/Abβ immunosensor allowed a wider dynamic range relevant to proteasome levels in serum. On the other hand, an ELISA method allowed the detection of proteasome in the same concentration range [32] as the proposed Au/4-MPBA/Abβ electrochemical immunosensor.

A comparison was also performed in relation to the only electrochemical immunosensor described in the scientific literature whose detection principle is based on the inhibition of the polymer electroactivity. Although this type of immunosensor allowed very low detection limits [18], its limited dynamic range makes difficult the precise detection of 20S in a relevant biological sample without intensive sample pretreatment.

## 4. Conclusions

This work described the development of an electrochemical immunosensor for the detection of 20S proteasome, a biomarker of some pathological conditions. The proposed immunosensor comprises the oriented immobilization of a capture antibody (Abβ) on a self-assembled monolayer of mercaptophenylboronic acid (4-MPBA) on gold electrodes, which can be fabricated in a very reproducible manner with low costs compared with the classical methods for 20S detection. Surface plasmon resonance and gravimetric analysis with quartz crystal microbalance demonstrated a high affinity of 20S for the immobilized Abβ.Scanning electron microscopy demonstrated a pattern of parallel lines upon immobilization of Abβ on 4-MPBA and morphological changes to a well-organized granular structure upon binding of 20S. A dual electrochemical detection strategy for 20S proteasome with the Au/4-MPBA/Abβ immunosensor was proposed.

The first methodology involved the correlation of 20S concentration with its proteolytic activity when bound to Abβ. The proteolysis of the Z-LLE-AMC substrate as a function of Abβ-bounded 20S was investigated with the Au/4-MPBA/Abβ/20S and used as an analytical signal for 20S detection with LoD = 1.4 µg mL^−1^ by fixed potential amperometry at +0.80 V (vs. Ag/AgCl). This methodology evidences not only the applicability of Au/4-MPBA/Abβ/20S for the quantification of 20S but also for the evaluation of 20S activity, which can be further developed into an assay for inhibitor screening.

The second methodology involved the correlation of 20S concentration with the enzymatic activity of an alkaline phosphatase-labeled secondary antibody (Ab_core_-AlkP) that binds on the 20S previously attached to the Abβ layer. The enzymatic activity of AlkP was investigated with the Au/4-MPBA/Abβ/20S/Ab_core_-AlkP, and the conversion of aminophenylphosphate to the electroactive aminophenol was used as an analytical signal for 20S detection with LoD = 0.2 µg mL^−1^ by fixed potential amperometry at +0.20 V (vs. Ag/AgCl).

Both strategies allowed the detection of 20S proteasome in the concentration range between 5 and 100 µg mL^−1^, which corresponds to proteasome levels in serum in the case of diverse pathological conditions. The proposed biosensor was applied to the detection of 20S in serum samples with recovery values ranging from 101 to 103%.

## Figures and Tables

**Table 1:** Values of equivalent circuit elements used to fit the spectra in Figure 3b.

Electrode Configuration	*R*_1_/ kΩ cm^2^	CPE_1_/ µF cm^−2^ s*^α^*^−1^	*α*_1_	*R*_2_/kΩ cm^2^	CPE_2_/ µF cm^−2^ s*^α^*^−1^	*α*_2_
Au	12	26	0.90	38	45	0.93
Au/4-MPBA	5	115	0.80	44	32	1.00
Au/4-MPBA/Abβ	13	55	0.76	320	22	0.95
Au/4-MPBA/Abβ/20S	10	55	0.80	360	22	0.97
Au/4-MPBA/Abβ/20S/Ab_core_-AlkP	11	65	0.76	620	20	0.92

**Table 2:** Comparison with other AlkP based electrochemical immunosensors.

Target Analyte	AlkP Substrate	Technique, *E*/V	Dynamic Range/ ng mL^−1^	LoD/µg mL^−1^	Ref.
α-Tumor Necrosis Factor	1-NP ^1^	DPV, +0.30	0.02–200	1.0 × 10^−5^	[38]
IgG	PAA ^2^	CA, +0.40	3–200	1.0 × 10^−3^	[24]
tetrodoxin	1-NP	DPV, +0.30	2–50	1.0 × 10^−3^	[37]
S and N proteins, of SARS-CoV2	1-NP	DPV, +0.20	-	1.9 × 10^−2^/ 0.8 × 10^−2^	[39]
Cancer biomarker	PAA	CV, +0.30	0.4–100	2.0 × 10^−4^	[35]
IL-1 beta cytokine	1-NP	DPV, +0.20	0.2–1.2	5.2 × 10^−6^	[40]
Cancer biomarker	3-IP ^3^	LSV, +0.15	5–50	2.8 × 10^−3^	[36]
20 S proteasome	AmPhP	CA, +0.20	5–100 × 10^3^	2.0 × 10^−1^	This work

^1^ Naphtyl phosphate, ^2^ phospho-ascorbic acid, ^3^ indoxyl phosphate.

**Table 3:** Comparison with other 20S detection methods.

Method		Dynamic Range/ µg mL^−1^	LoD/ µg mL^−1^	Ref.
SPR		1.0–15.0	-	[12]
SPR	Chip 1	1.5–7.5	1.2 × 10^−1^	[11]
	Chip 2		2.0 × 10^−1^	
ELISA KIT		0.5–20.0	-	[32]
Electrochemical		0.1–0.2	1.0 × 10^−2^	[18]
Electrochemical		5.0–100.0	2.0 × 10^−1^	this work

## Data Availability

The datasets generated and analyzed during the current study are available from the corresponding author on reasonable request.

## Supplementary Materials

The following are available online at https://www.mdpi.com/article/10.3390/s21082702/s1, Figure S1: Electrochemical detection of AmPh at an Au electrode by (a) Cyclic voltammetry and (b) fixed potential CA at +0.2 V vs. Ag/AgCl and 2-corresponding calibration plots, Table S1: Analytical parameters calculated for the Au/4-MPBA/Abβ/20S using the linear fit of the curves in Figure 3. RSD obtained from 3 independent experiments, Table S2: Fitting parameters obtained for the linear dependence of *S*_Z-LLE-AMC_ and *j_75_* and in Figure 4c vs. log*[20S]*. Table S3: Analytical parameters calculated for the Au/4-MPBA/Abβ/20S/Ab_core_-AlkP for various concentrations of 20S using the linear fit of the curves in Figure 4. RSD obtained from 3 independent experiments, Table S4: Kinetic parameters calculated for the Au/4-MPBA/Abβ/20S/Ab_core_-AlkP for various concentrations of 20S using the Michaelis Menten non-linear fit of the curves in Figure 4. RSD obtained from 3 independent experiments, Table S5: Fitting parameters obtained for the linear dependence of *j*_max_ and *S*_AmPhP_ in Figure 5D vs. log*[20S]*.
