# Supplementary material for: Immobilized Antibodies on Mercaptophenylboronic Acid Monolayers for Dual-Strategy Detection of 20S Proteasome
Source: Sensors (Basel). 2021 Apr 12;21(8):2702. doi: 10.3390/s21082702 (PMC8068791; doi:10.3390/s21082702)
Supplement: Supplementary file 1 [file sensors-21-02702-s001.pdf]

sensors

MDPI

Supplementary Information

Immobilized Antibodies on Mercaptophenylboronic Acid Monolayers for Dual-Strategy Detection of 20S Proteasome

Madalina M. Barsan [1], Caroline G. Sanz [1], Melania Onea[1,2] and Victor C. Diculescu [1,*]

[1] National Institute of Materials Physics, Atomistilor 405A, 077125 Măgurele, Romania; madalina.barsan@infim.ro (M.M.B.); caroline.sanz@infim.ro (C.G.S.); melania.onea@infim.ro (M.O.)

[2] Faculty of Physics, University of Bucharest, Atomistilor 405, 077125 Măgurele, Romania

* Correspondence: victor.diculescu@infim.ro; Tel.: +40-213690185

1. Figures

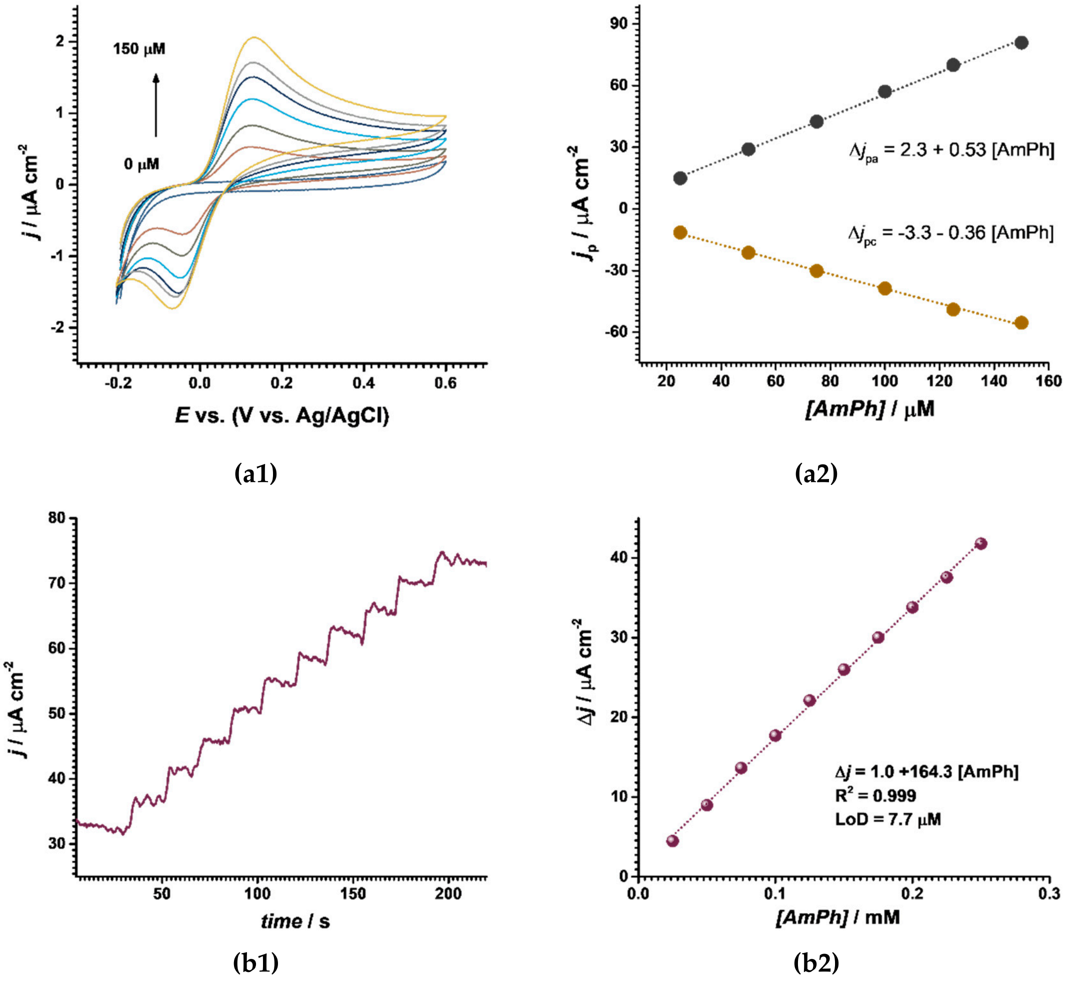

Figure 1. Electrochemical detection of AmPh at an Au electrode by (**a**) Cyclic voltammetry and (**b**) fixed potential CA at +0.2 V vs. Ag/AgCl and (1) and (2) corresponding calibration plots.

2. Tables

Table 1. Analytical parameters calculated for the Au/4-MPBA/Abβ/20S using the linear fit of the curves in Figure 3. RSD obtained from 3 independent experiments.

[20S] / μg mL^{-1}	$S_{Z\text{-}LLE\text{-}AMC}$ / nA cm^{-2} mM^{-1}	RSD / %	R^2	LOD / μM
0.05	18.9 ± 1.3	6.6	0.995	5.17
0.5	39.1 ± 2.8	7.1	0.984	9.15
5	51.4 ± 3.3	6.5	0.997	3.71
50	73.2 ± 5.1	7.0	0.997	4.10
100	94.4 ± 6.4	6.8	0.998	3.18
250	127.6 ± 8.8	6.9	0.999	2.37
1000	160.1 ± 10.9	6.8	0.999	1.82

Table 2. Fitting parameters obtained for the linear dependence of $S_{Z\text{-}LLE\text{-}AMC}$ and j_{75} and in Figure 4c vs. log*[20S]*.

parameter	*[20S]* / μg mL^{-1}	*sensitivity* /	LoD / μg mL^{-1}
$S_{Z\text{-}LLE\text{-}AMC}$	0.05–5	16.2 ± 2.2 nA cm^{-2} mM^{-1} (μg mL^{-1})$^{-1}$	0.60
	50–1000	67.5 ± 4.6 nA cm^{-2} mM^{-1} (μg mL^{-1})$^{-1}$	0.20
j_{75}	0.05–5	1.26 ± 0.15 nA cm^{-2} (μg mL^{-1})$^{-1}$	2.11
	50–1000	4.45 ± 0.13 nA cm^{-2} (μg mL^{-1})$^{-1}$	0.11

Table 3. Analytical parameters calculated for the Au/4-MPBA/Abβ/20S/Ab$_{core}$-AlkP for various concentrations of 20S using the linear fit of the curves in Figure 4. RSD obtained from 3 independent experiments.

[20S] / μg mL^{-1}	S_{AmPhP} / nA cm^{-2} mM^{-1}	RSD / %	R^2	LOD / μM
0.05	200.7 ± 17.6	8.8	0.997	4.77
0.5	209.5 ± 16.3	7.8	0.998	3.18
5	228.8 ± 16.9	7.4	0.984	10.65
50	362.7 ± 28.0	8.0	0.982	7.16
100	509.3 ± 48.2	8.2	0.997	1.88
250	630.9 ± 54.9	7.8	0.996	2.76
1000	821.9 ± 64.3	8.2	0.981	3.18

Table 4. Kinetic parameters calculated for the Au/4-MPBA/Abβ/20S/Ab$_{core}$-AlkP for various concentrations of 20S using the Michaelis Menten non-linear fit of the curves in Figure 4. RSD obtained from 3 independent experiments.

[20S] / μg mL^{-1}	j_{max} / nA cm^{-2}	K_M / mM	R^2
0.05	279.4 ± 14.4	1.11 ± 0.09	0.994
0.5	325.6 ± 15.6	1.22 ± 0.08	0.998
5	397.5 ± 18.8	1.23 ± 0.08	0.997
50	721.9 ± 16.3	1.52 ± 0.08	0.997
100	885.6 ± 20.0	1.28 ± 0.1	0.997
250	1063.0 ± 54.1	1.20 ± 0.1	0.996
1000	1405.0 ± 15.6	1.30 ± 0.09	0.995

Table 5. Fitting parameters obtained for the linear dependence of j_{max} and S_{AmPhP} in Figure 5D vs. log*[20S]*.

**parameter**	***[20S]* / μg mL^{-1}**	***sensitivity* /**	**LoD / μg mL^{-1}**
j_{max}	0.05–5	59.1 ± 7.2 nA cm^{-2} (μg mL^{-1})$^{-1}$	0.53
	50–1000	520.0 ± 17.1 nA cm^{-2} (μg mL^{-1})$^{-1}$	0.10
S_{AmphP}	0.05–5	14.1 ± 3.0 nA cm^{-2} mM^{-1} (μg mL^{-1})$^{-1}$	0.91
	50–1000	344.1 ± 24.1 nA cm^{-2} mM^{-1} (μg mL^{-1})$^{-1}$	0.20
